# Oculopharyngeal Muscular Dystrophy as a Paradigm for Muscle Aging

**DOI:** 10.3389/fnagi.2014.00317

**Published:** 2014-11-10

**Authors:** Yotam Raz, Vered Raz

**Affiliations:** ^1^Department of Human Genetics, Leiden University Medical Center, Leiden, Netherlands

**Keywords:** adult myopathy, muscle degeneration, OPMD, PABPN1, RNA metabolism

## Abstract

Symptoms in late-onset neuromuscular disorders initiate only from midlife onward and progress with age. These disorders are primarily determined by identified hereditable mutations, but their late-onset symptom manifestation is not fully understood. Here, we review recent research developments on the late-onset autosomal dominant oculopharyngeal muscular dystrophy (OPMD). OPMD is caused by an expansion mutation in the gene encoding for poly-adenylate RNA binding protein1 (PABPN1). The molecular pathogenesis for the disease is still poorly understood. Despite a ubiquitous expression of PABPN1, symptoms in OPMD are limited to skeletal muscles. We discuss recent studies showing that PABPN1 levels in skeletal muscles are lower compared with other tissues, and specifically in skeletal muscles, PABPN1 expression declines from midlife onward. In OPMD, aggregation of expanded PABPN1 causes an additional decline in the level of the functional protein, which is associated with severe muscle weakness in OPMD. Reduced PABNPN1 expression in muscle cell culture causes myogenic defects, suggesting that PABPN1 loss-of-function causes muscle weakness in OPMD and in the elderly. Molecular signatures of OPMD muscles are similar to those of normal muscle aging, although expression trends progress faster in OPMD. We discuss a working hypothesis that aging-associated factors trigger late-onset symptoms in OPMD, and contribute to accelerated muscle weakness in OPMD. We focus on the pharyngeal and eyelid muscles, which are often affected in OPMD patients. We suggest that muscle weakness in OPMD is a paradigm for muscle aging.

In aging populations, late-onset disorders are highly common, among which late-onset neuromuscular (NM) disorders are a subset. NM disorders affect muscle fibers and/or the central and peripheral nervous system and the NM junction that control the muscle fibers. At present, these disorders are often incurable. As life expectancy rises, the prevalence of late-onset disorders causing chronic muscle weakness increases. Muscle symptoms can manifest from midlife onward, leading to a drastic functional decline with social and economic burdens. This suggests that in addition to the fundamental genetic defect(s), possible similar aging-associated regulators trigger the late onset of symptoms and progression thereof. For example, protein catabolism, which discards defective or redundant proteins (mainly through the ubiquitin proteasome and autophagy systems), has been implicated as a predominant regulator in normal aging and late-onset diseases [reviewed in Low (2011) and Bonaldo and Sandri (2013)]. Reduced protein catabolism can also lead to an accumulation of aggregation-prone proteins and formation of insoluble protein aggregates. These aggregates are often the pathological hallmark in a number of late-onset neurological and/or muscular disorders (Ruegg and Glass, 2011; Bonaldo and Sandri, 2013). Although symptoms of these diseases widely vary, symptoms often initiate in a small subset of muscular or neuronal tissues (Ross and Poirier, 2005). While the primary genetic causes for these disorders are known, why symptoms initiate from midlife onward in specific tissues and how symptoms progress with age is still obscure.

Oculopharyngeal muscular dystrophy (OPMD) is an autosomal dominant and rare myopathy. The estimated prevalence in Western countries is 1:100,000 [reviewed in Raz et al. (2013)]. Due to founder effects, clusters with a higher prevalence are found in French-Canadians and in the Bukhara community in Israel (1:1000 and 1:600, respectively) (Blumen et al., 2000; Laberge et al., 2005). It has been suggested, however, that outside these communities, the disease remains underdiagnosed (Ruegg et al., 2005). In OPMD, skeletal muscles are predominantly affected, whereby initial symptoms are manifested in only a subset of muscles. Most commonly, this leads to lowering (ptosis) of the eyelids and swallowing difficulties (dysphagia). With disease progression, additional skeletal muscles can be affected including the proximal muscles of the lower limb (including the quadriceps muscles) (Fischmann et al., 2012). OPMD is a monogenic disorder and its etiology is found in an alanine expansion mutation in the gene encoding for poly-adenylate (poly(A)) binding protein nuclear 1 (PABPN1) (Brais et al., 1998). Formation of insoluble inclusions in the cell nucleus is the pathological hallmark of OPMD muscles (Tome and Fardeau, 1980). Under physiological expression levels, expanded (exp)PABPN1 is more prone to aggregation compared with the wild-type PABPN1 (Raz et al., 2011a). High overexpression of expPABPN1 in muscles of various animal models causes muscle weakness, and it is suggested that accumulation of aggregates could be the cause for the muscle dysfunction (Davies et al., 2005). High overexpression of expPAPBN1 in animal models, as well as in cellular models leads to cell death, suggesting that expPABPN1 aggregates are toxic (Davies et al., 2008). Importantly, treatments that may reduce aggregation lead to less cell death and reduced muscle weakness in these animal models (Davies et al., 2006, 2010; Catoire et al., 2008; Chartier et al., 2009). Based on those models, it was suggested that muscle symptoms in OPMD are caused by a PABPN1 gain-of-function. However, it is not resolved whether animal models with high overexpression in muscles are relevant to OPMD. For example, high overexpression of expPABPN1 was not reported in OPMD heterozygous patients. Moreover, aggregates of wild-type PABPN1 were found in unaffected rat neural cells (Berciano et al., 2004). Altogether, it is striking that despite the well-known genetic cause for OPMD, the molecular mechanisms and physiological conditions that lead to muscle symptoms are poorly understood. Here, we discuss four questions for OPMD pathophysiology.

## Is OPMD an RNA Metabolism Disorder?

Poly-adenylate RNA binding protein1 is multifunctional regulator of RNA metabolism. Initially, it was identified *in vitro* as a regulator of poly(A) tail length (Kerwitz et al., 2003), subsequently was validated *in vivo* (Benoit et al., 2005), and more recently, it was shown to have an impact on mRNA decay (Bresson and Conrad, 2013). PABPN1 knockdown in mouse muscle cells causes reduced poly(A) tail length that is associated with myogenesis defects (Apponi et al., 2010); however, the relevance for OPMD and muscle aging is unsettled. A change in poly(A) tail length was not found in muscles from OPMD patients (Calado et al., 2000). Recent studies revealed additional molecular functions for PABPN1. A genome-wide shift from distal to proximal alternative polyadenylation site (PAS) and accumulation of shortened transcripts were found in the mouse model for OPMD, A17.1, which was generated by expPABPN1 overexpression in muscles, and in cells with reduced PABPN1 expression (de Klerk et al., 2012; Jenal et al., 2012). Similar alternative PAS utilization was found in models with expPABPN1 overexpression and PABPN1 downregulation, suggesting that PABPN1 loss-of-function causes defects in RNA metabolism (de Klerk et al., 2012; Jenal et al., 2012). In OPMD muscles, PABPN1 downregulation was found to be comparable to age-matching controls (Anvar et al., 2013). Reduced PABPN1 levels in cellular models cause myogenic defects (Apponi et al., 2010; Anvar et al., 2013). In addition, PABPN1 was also found to regulate long non-coding RNA expression (Beaulieu et al., 2012). However, so far, alternative PAS or long non-coding RNA expression was not reported in OPMD patient muscles. To adequately understand how PABPN1 regulates changes in RNA metabolism in OPMD with an impact on muscle weakness, experiments should be conducted in models with physiological levels of PABPN1.

Aberrant RNA metabolism is not specific to OPMD, but is found in a wide spectrum of unrelated late-onset neurological and/or muscular disorders (O’Rourke and Swanson, 2009; Anthony and Gallo, 2010). Since these disorders share a late onset of symptoms and progression with age, age-associated regulators of RNA metabolism could be affected. It is still unclear whether similar regulators of RNA metabolism are affected in these disorders. In OPMD, affected muscles as well as muscles from pre-symptomatic family members can be accessible for research (Fischmann et al., 2012; Anvar et al., 2013). Therefore, OPMD could be used as a paradigm to study the functional contribution of RNA metabolism to symptoms in late-onset neurological and/or muscular disorders and to study a possible regulatory role in the age-associated progression of the symptoms.

## Why are Skeletal Muscles Primarily Affected in OPMD?

Poly-adenylate RNA binding protein1 is essential for cell vitality (Bhattacharjee and Bag, 2012) and is expressed in all cells; however symptoms are predominantly manifested in skeletal muscles. In OPMD patients, PABPN1 expression is specifically reduced in affected *Vastus lateralis* muscles, while PABPN1 levels in whole blood are unchanged between OPMD patients and healthy controls (Anvar et al., 2013). In Dutch and Danish OPMD patients, weakness of the quadriceps muscles is reported as one of the initial symptoms (Sluijs et al., 2003; Witting et al., 2014). PAPBN1 level is lower in skeletal muscles of both human being and mouse, compared with a spectrum of tissues (Apponi et al., 2013). Since symptoms in OPMD are predominant in skeletal muscles, this suggests that below a certain crucial level, a functional impact is manifested. Although a threshold for functional PABPN1 is yet to be defined, due to low PABPN1 expression levels in skeletal muscles (Apponi et al., 2013) this crucial level could reach a functional impact. In other tissues, however, PABPN1 levels are sufficiently high (Apponi et al., 2013), and thus, if any aging-associated decline may occur (Anvar et al., 2013), they would be spared from a functional deficiency (Figure 2). Indeed, altered PABPN1 at 40, 60, or 80% expression level causes reciprocal decrease in the expression of sarcomeric genes (Anvar et al., 2013). This working model requires additional *in vivo* experiments.

**Figure 1:**
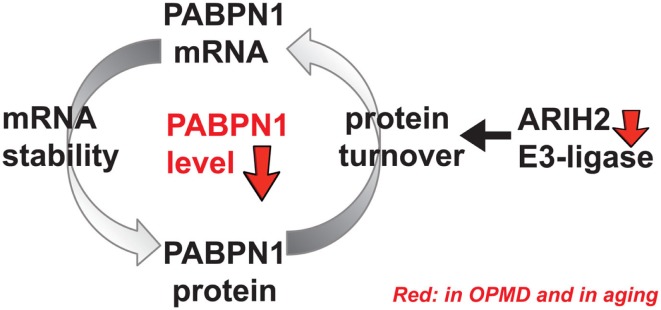
**A schematic working model for regulation of PABPN1 levels**. PABPN1 levels are established by a feed-forward loop combining at least regulation of mRNA stability and protein turnover. PABPN1 protein accumulation is regulated by the ubiquitin proteasome system (UPS), including ARIH2 E3-ligase. An aging-associated distortion of the UPS causes impairment of PABPN1 protein turnover affecting its mRNA stability and genes of the UPS, including ARIH2 E3-ligase. In turn, PABPN1 mRNA levels, which are self-regulated, also decrease with aging. However, the molecular factors regulating PABPN1 mRNA stability are unknown as yet. In OPMD and in aging, reduced levels of PABPN1 eventually cause genome-wide changes in gene expression.

It is not fully understood why levels of PABPN1 are lower in skeletal muscles. PABPN1 mRNA is less stable in muscles compared with other tissues (Apponi et al., 2013). PABPN1 mRNA binds to PABPN1 protein (Raz et al., 2014), which potentially affects PABPN1 mRNA stability, nuclear export, and translation (Figure 1). As yet, regulators of mRNA stability in muscles and aging-associated changes are poorly understood. In addition, PABPN1 protein turnover is regulated by the ubiquitin proteasome system (UPS) (Raz et al., 2011b). Differences in poly-ubiquitination levels between wild type and expPABPN1 result in higher protein turnover of wild-type PAPBN1 as compared with expPABPN1 (Raz et al., 2011b). Since PABPN1 is prone to aggregation, higher protein accumulation leads to aggregate formation and reduced availability of the functional protein. PABPN1 protein accumulation is regulated by ARIH2 E3-ligase, whose level also declines from midlife onward in skeletal muscles (Raz et al., 2014). In OPMD muscles, ARIH2 level is lower compared to age-matching controls, which in part could result by the alternative PAS utilization in ARIH2 3′-UTR that is directly regulated by PABPN1 level. In addition, as ARIH2 protein is entrapped in expPABPN1 aggregates, functional protein levels would be depleted (Raz et al., 2014). Protein entrapment in PABPN1 aggregates was reported for other E3 ligases and the proteasome (Corbeil-Girard et al., 2005; Tavanez et al., 2005; Anvar et al., 2011), suggesting that the UPS machinery is dysregulated in OPMD. In turn, mRNA dysregulation of the UPS was found to be the most consistent dysregulated cellular system in OPMD (Anvar et al., 2011; de Klerk et al., 2012). Although a mechanistic model for regulation of PABPN1 levels in aging and muscle disease calls for additional research, the current literature suggests that changes in PABPN1 levels in muscles are regulated by both mRNA stability and protein turnover and that these machineries are specifically affected in OPMD (Figure 1). Regulators of PABPN1 mRNA stability and protein turnover should be identified in future studies.

## Why Specific Muscles Seem to be More Affected than Other Muscles?

In OPMD, the ocular and pharyngeal muscles are initially the most commonly affected; however, with progression of the disease, more skeletal muscles are affected. These muscles are also often affected in otherwise healthy elderly. Most skeletal muscles are weakened during aging; however, it is unclear whether aging-associated muscle weakness is programed, concerning initially affected muscles and progression. Relevant to OPMD, dysphagia, resulting from weakness of pharyngeal muscles, and eyelid ptosis, resulting from weakness of the *levator palpebrae* muscle, is highly common in the elderly (Salvi et al., 2006; Fea et al., 2013; Iida et al., 2013). In contrast to the otherwise healthy population, in OPMD, symptoms start at an earlier age and seem more severe, possibly due to faster progression. Eyelid ptosis can also be caused by rare multiple mitochondrial DNA deletion syndromes, including progressive external ophthalmoplegia (PEO) (Van Goethem et al., 2003). Increased mitochondrial deletions were found in the extra-ocular muscles compared to other muscles and are aging associated (Yu-Wai-Man et al., 2010), indicating a prominent role for mitochondrial activity in ocular muscles during aging. A decrease in mitochondrial activity was reported in a mouse model for OPMD (Trollet et al., 2010) and in a muscle cell model with PABPN1 downregulation (Anvar et al., 2013). These studies suggest that in OPMD, mitochondrial dysfunction may contribute to the muscle weakness and future studies should explore a role for PAPBN1 in the regulation of mitochondrial genes and mitochondrial and metabolic cellular functionality.

Dysphagia in the elderly is determined by age-associated anatomical changes in the neck and throat, underpinning the swallowing function in otherwise healthy elderly (Ney et al., 2009). Furthermore, dysphagia is also common in different age-related diseases including NM disorders (Schindler and Kelly, 2002). Dysphagia in the otherwise healthy elderly manifests by a less efficient passage of food to the esophagus due to changes in swallowing pressures of the upper esophageal sphincter and delayed muscle movements (Yokoyama et al., 2000). Symptoms are suggested to be associated with an increase in muscle atrophy and a decrease in muscle strength of the pharyngeal muscles (Feng et al., 2013).

Although it is not clear why ocular and pharyngeal muscles are initially affected in OPMD, the ocular and pharyngeal muscles are constantly, and often unconsciously, routinely used in daily life. Interestingly, PABPN1 levels in mice pharyngeal muscle are lower compared with other skeletal muscles (Apponi et al., 2013). This suggests that this muscle could be more susceptible to a further decrease in PABPN1 levels. However, functional studies, especially in human beings, would lead to conclusive answers.

## Why Symptoms Initiate from Midlife Onward and Does OPMD Represent Accelerated Muscle Aging?

Muscle aging (sarcopenia) is marked, among others, by a decrease in muscle mass (atrophy), an increase in fat infiltration and inflammation leading to a decrease in muscle strength and physical performance (Cruz-Jentoft et al., 2010). In the A17.1 mouse model, muscle atrophy appears already from 12 weeks of age (24–month-old mice are considered as aged) and is complemented by expression dysregulation of known muscle atrophy genes such as Murf1 and Atrogin-1 (Trollet et al., 2010). Muscle atrophy in this mouse model is prominent in the fast glycolytic (type IIX/IIB) fibers, whereas the slow oxidative (type I) and fast oxidative (type IIA) fibers seem to be spared (Trollet et al., 2010). Although it is widely accepted that aged muscles of rodents are enriched in slow (type I) fibers compared to fast (type II) fibers, in aging human muscles, the literature concerning this fiber-type switch is not conclusive (Purves-Smith et al., 2014). Therefore, the implications of the muscle fiber-type switch for OPMD patients are unsettled. Studies in additional models are necessary in order to reveal whether reduced PABPN1 levels induce muscle atrophy and muscle fiber-type switch. Muscle aging can also be non-invasively quantified from magnetic resonance imaging (MRI) including muscle cross-sectional surface area, fatty infiltration (Fischmann et al., 2012; Willis et al., 2013), and inflammation (Eshed et al., 2007). These measures can be considered as approximations of muscular quality. With these measures, progressive muscular atrophy in OPMD was documented (Fischmann et al., 2012).

**Figure 2:**
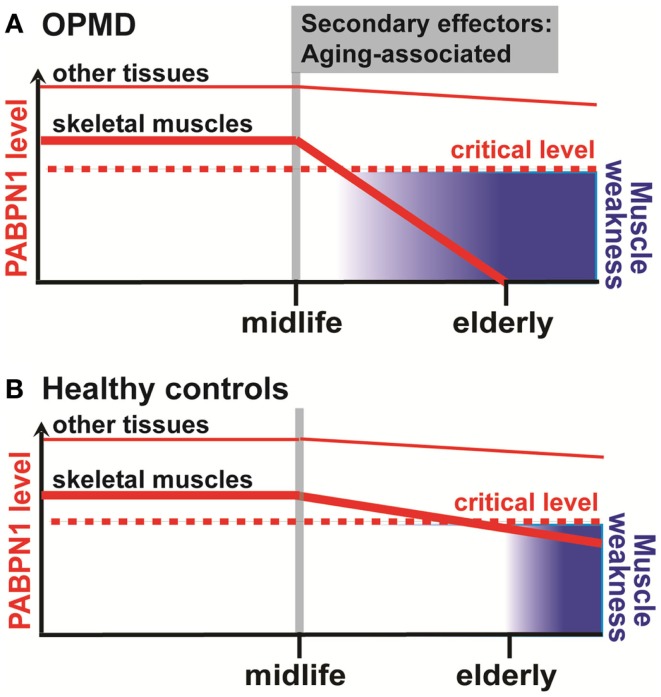
**A schematic working model for the aging-associated decline of muscle strength and PABPN1 in OPMD (A) and healthy controls (B)**. In skeletal muscles, a decline in PABPN1 levels starts from midlife onward in both OPMD and healthy controls. The onset of decline from midlife onward is caused by secondary age-associated effectors, which mostly are unknown. Eventually, below a critical PABPN1 level, muscle weakness symptoms are manifested. This process is accelerated in OPMD due to aggregation of expanded PABPN1, which further depletes levels of functional PABPN1. In other tissues, however, levels of PABPN1 are higher and a possible age-related decline of PABPN1 does not reach the critical level for a functional impact.

Although OPMD is often grouped within the muscular dystrophies, our studies of RNA expression profiles in OPMD muscles and OPMD models revealed higher similarities with muscles from the elderly rather than with muscular dystrophies or myopathies (Anvar et al., 2013), suggesting similar molecular signatures. Overlapping dysregulated genes were found for the mitochondria, the UPS, DNA repair, TGF-β signaling, and sarcomeric genes (Anvar et al., 2013). However, functional studies should investigate a role for additional aging-associated pathways. For example, the autophagy system has also been found to have an imperative regulatory role in many age-associated diseases, in healthy aging (Schneider and Cuervo, 2014), and in muscle atrophy (Bonaldo and Sandri, 2013). As PABPN1 can shuttle between the nucleus and the cytoplasm (Abu-Baker et al., 2005; Benoit et al., 2005), it should be investigated whether PABPN1 is also regulated by the autophagy system. Moreover, genes of the autophagy system could be regulated by PABPN1 (Anvar et al., 2013). More interestingly, age-associated changes in a cross-sectional data revealed a faster change in expression level for a subset of genes, among which many are known as aging genes, muscle-specific sarcomeric genes, and PABPN1 (Anvar et al., 2013). This suggests that muscle weakness in OPMD could represent accelerated muscular aging, and thus, OPMD muscles could be a paradigm for otherwise healthy muscle aging (Figure 2).

## Conflict of Interest Statement

The authors declare that the research was conducted in the absence of any commercial or financial relationships that could be construed as a potential conflict of interest.
